# Learn Quasi-Stationary Distributions of Finite State Markov Chain

**DOI:** 10.3390/e24010133

**Published:** 2022-01-17

**Authors:** Zhiqiang Cai, Ling Lin, Xiang Zhou

**Affiliations:** 1 School of Data Science, City University of Hong Kong, Tat Chee Ave, Kowloon, Hong Kong, China; xiang.zhou@cityu.edu.hk; 2 School of Mathematics, Sun Yat-sen University, Guangzhou 510275, China; linling27@mail.sysu.edu.cn; 3 Department of Mathematics, City University of Hong Kong, Tat Chee Ave, Kowloon, Hong Kong, China

**Keywords:** quasi-stationary distribution, reinforcement learning, KL-divergence, actor-critic algorithm

## Abstract

We propose a reinforcement learning (RL) approach to compute the expression of quasi-stationary distribution. Based on the fixed-point formulation of quasi-stationary distribution, we minimize the KL-divergence of two Markovian path distributions induced by candidate distribution and true target distribution. To solve this challenging minimization problem by gradient descent, we apply a reinforcement learning technique by introducing the reward and value functions. We derive the corresponding policy gradient theorem and design an actor-critic algorithm to learn the optimal solution and the value function. The numerical examples of finite state Markov chain are tested to demonstrate the new method.

## 1. Introduction

Quasi-stationary distribution (QSD) is the long time statistical behavior of a stochastic process that will be surely killed when this process is conditioned to survive [1]. This concept has been widely used in applications, such as in biology and ecology [2,3], chemical kinetics [4,5], epidemics [6,7,8], medicine [9] and neuroscience [10,11]. Many works for rare events in meta-stable systems also focus on this quasi-stationary distribution [12,13]. In addition, some new Monte Carlo sampling methods, for instance, the Quasi-stationary Monte Carlo method [14,15], also arise by using QSD instead of true stationary distribution, for instance, the Quasi-stationary Monte Carlo method [14,15]

We are interested in the numerical computation of QSD and focus on the finite state Markov chain in this paper. Mathematically, the quasi-stationary distribution can be solved as the principal left eigenvector of a sub-Markovian transition matrix. Thus, traditional numerical algebra methods can be applied to solve the quasi-stationary distribution in finite state space, for example, the power method [16], the multi-grid method [17] and Arnoldi’s algorithm [18]. These eigenvector methods can produce a stochastic vector for QSD instead of generating samples of QSD.

In search of efficient algorithms for large state space, stochastic approaches are in favor of either sampling the QSD or computing the expression of QSD, and these methods can be applied or extended easily to continuous state space. A popular approach for sampling quasi-stationary distribution is the Fleming–Viot stochastic method [19]. The Flemming–Viot method first simulates *N* particles independently. When any one of the particles falls into the absorbing state and becomes killed, a new particle is uniformly selected from the remaining N−1 surviving particles to replace the dead one, and the simulation continues. When time and *N* tend to infinity, the particles’ empirical distribution can converge to the quasi-stationary distribution.

In [20,21,22], the authors proposed to recursively update the expression of QSD at each iteration based on the empirical distribution of a single-particle simulation. It is shown in [21] that the convergence rate can be $O(n^{-1/2})$, where *n* is the iteration number. This method is later improved in [23,24] by applying the stochastic approximation method [25] and the Polyak–Ruppert averaging technique [26]. These improved algorithms have a choice of flexible step size but require a projection operator onto probability simplex, which carries some extra computational overhead increasing with the number of states. Ref. [15] extended the algorithm to the diffusion process.

In this paper, we focus on how to compute the expression of the quasi-stationary distribution, which is denoted by α(x) on a metric space E. If E is finite, α is a probability vector, and if E is a domain in $R^{d}$, then α is a probability density function on E. We assume α can be numerically represented in parametric form $\alpha_{\theta }$ and θ∈Θ. This family $\left\{\alpha_{\theta }\right\}$ can be in tabular form or any neural network. Then, the problem of finding the QSD α becomes answering the question of how to compute the optimal parameter θ in Θ. We call this problem the learning problem for QSD. In addition, we want to directly learn QSD and not use the distribution family $\left\{\alpha_{\theta }\right\}$ to fit the simulated samples generated by other traditional simulation methods.

Our minimization problem for QSD is similar to the variational inference (VI) [27], which minimizes an objective functional measuring the distance between the target and candidate distributions. However, unlike the mainstream VI methods such as evidence lower bound (ELBO) technique [28] or particle-based [29], flow-based methods [30], our approach is based on recent important progresses from reinforcement learning (RL) method [31], particularly the policy gradient method and actor-critic algorithm. We first regard the learning process of the quasi-stationary distribution as the interaction with the environment, which is constructed by the property of QSD. Reinforcement learning has recently shown tremendous advancements and remarkable successes in applications (e.g., [32,33,34]). The RL framework provides an innovative and powerful modeling and computation approach for many scientific computing problems.

The essential question is how to formulate the QSD problem as an RL problem. Firstly, for the sub-Markovian kernel *K* of a Markov process, we can define a Markovian kernel $K_{\alpha }$ on E (see Definition 1) and then QSD is defined by the equation $\alpha =\alpha K_{\alpha }$, which equals α as the initial distribution and the distribution after one step. Secondly, we consider an optimal α (in our parametric family of distribution) to minimize the Kullback–Leibler divergence (i.e., relative entropy) of two path distributions, denoted by P and Q, associated with two Markovian kernels $K_{\alpha }$ and $K_{\beta }$ where $\beta :=\alpha K_{\alpha }$. Thirdly, inspired by the recent work [35] of using RL for rare events sampling problems, we transform the minimization of KL divergence between P and Q into the maximization of a time-averaged reward function and defined the corresponding value function V(x) at each state *x*. This completes our modeling of RL for the quasi-stationary distribution problem. Lastly, we derive the policy gradient theorem (Theorem 1) to compute the gradient with respect to θ of the averaged reward for the learning dynamic for the averaged reward. This is known as the “actor” part. The “critic” part is to learn the value function *V* in its parametric form $V_{\psi }$. The actor-critic algorithm uses the stochastic gradient descent to train the parameter θ for the action $\alpha_{\theta }$ and the parameter ψ for the value function $V_{\psi }$ (see Algorithm 1).

Our contribution is that we first devise a method to transform the QSD problem into the RL problem. Similar to [35], our paper also uses the KL-divergence to define the RL problem. However, our paper fully adapts the unique property of QSD that is a fixed point problem $\alpha =\alpha K_{\alpha }$ to define the RL problem.

Our learning method allows the flexible parametrization of the distributions and uses the stochastic gradient method to train the optimal distribution. It is easy to implement optimization with scale up to large state spaces. The numerical examples we tested have shown our that methods converge faster than other existing methods [22,23].

Finally, we remark that our method works very well for QSD of the strict sub-Markovian kernel *K* but is not applicable to compute the invariant distribution when *K* is Markovian. This is because we transform the problem into the variational problem between two Markovian kernels $K_{\alpha }$ and $K_{\beta }$ (where $\beta =\alpha K_{\alpha }$). Note that $K_{\alpha }\left(x,y\right)=K\left(x,y\right)+\left(1-K\left(x,E\right)\right)\alpha \left(y\right)$ (Definition 1), and our method is based on the fact that α=β if and only if $K_{\alpha }=K_{\beta }$. If *K* is Markovian kernel, then $K_{\alpha }\equiv K$ for any α, and our method cannot work. Thus, K(x,E) has to be strictly less than 1 for some x∈E.

This paper is organized as follows. Section 2 is a short review of the quasi-stationary distribution and some basic simulation methods of QSD. In Section 3, we first formulate the reinforcement learning problem by KL-divergence and derive the policy gradient theorem (Theorem 1). Using the above formulation, we then develop the actor-critic algorithm to estimate the quasi-stationary distribution. In Section 4, the efficiency of our algorithms is illustrated by four examples compared with the simulation methods in [24].

**Algorithm 1:** (**ac-α method**) Actor-critic algorithm for quasi-stationary distribution $\alpha_{\theta }$

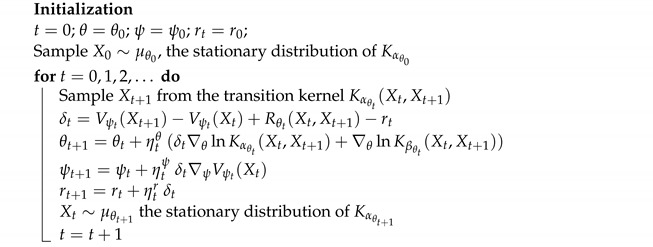



## 2. Problem Setup and Review

### 2.1. Quasi-Stationary Distribution

We start with an abstract setting. Let E be a finite state equipped with the Borel σ-field B(E), and let P(E) be the space of probabilities over E. A sub-Markovian kernel on E is defined as a map K:E×B(E)↦[0,1] such that for all x∈E,A↦K(x,A) is a nonzero measure with K(x,E)≤1 and for all A∈B(E),x↦K(x,A) is measurable. In particular, if K(x,E)=1 for all x∈E, then *K* is called a Markovian kernel. Throughout the paper, we assume that *K* is strictly sub-Markovian, i.e., K(x,E)<1 for some *x*.

Let $X_{t}$ be a Markov chain with values in $E\cup \left\{\partial \right\}$ where ∂∉E denotes an absorbing state. We define the extinction time

$$\tau :=\inf\left\{t>0:\;X_{t}=\partial \right\}.$$

We define the **quasi-stationary distribution (QSD)** α as the long time limit of the conditional distribution, if there exists a probability distribution ν on E such that the following is the case:

(1) $$\alpha \left(A\right):=\lim_{t\to \infty }P_{\nu }\left(X_{t}\in A|\tau >t\right),\;A\in B\left(E\right).$$

where $P_{\nu }$ refers to the probability distribution of $X_{t}$ associated with the initial distribution ν on E. Such a conditional distribution well describes the behavior of the process before extinction, and it is easy to see that α satisfies the following fixed point problem:

(2) $$P_{\alpha }\left(X_{t}\in A|\tau >t\right)=\alpha \left(A\right)$$

where $P_{\alpha }$ refers to the probability distribution of $X_{t}$ associated with the initial distribution α on E. Equation (2) is equivalent to the following stationary condition such that the following is the case:

(3) $$\alpha =\frac{\alpha K}{\alpha K1},\;\mathrm{or}\;\alpha \left(y\right)=\frac{\sum_{x}\alpha \left(x\right)K\left(x,y\right)}{\sum_{x}\alpha \left(x\right)K\left(x,E\right)}$$

where α is a row vector and 1 denotes the column vector with all entries being one and

$$K\left(x,E\right)=\sum_{x^{\prime }\in E}K\left(x,x^{\prime }\right).$$

For any sub-Markovian kernel *K*, we can associate *K* with a Markovian kernel $\tilde{K}$ on E∪{∂} defined by the following:

$$\left\{\begin{array}{c} \tilde{K}\left(x,A\right)=K\left(x,A\right)\\ \tilde{K}\left(x,\left\{\partial \right\}\right)=1-K\left(x,E\right)\\ \tilde{K}\left(\partial ,\left\{\partial \right\}\right)=1. \end{array}\right.$$

for all x∈E,A∈B(E). The kernel $\tilde{K}$ can be understood as the Markovian transition kernel of the Markov chain $\left(X_{t}\right)$ on E∪{∂} for which its transitions in E is specified by *K*, but it is “killed” forever once it leaves E.

In this paper, we assume E is a finite state space and the process in consideration has a unique QSD. Assume that *K* is irreducible, then existence and uniqueness of the quasi-stationary distribution can be obtained by the Perron–Frobenius theorem [36].

An important Markovian kernel is the following $K_{\alpha }$, which is defined on E only and has a “regenerative probability” α.

**Definition** **1.** *For any given α∈P(E) and a sub-Markovian kernel K on E, we define $K_{\alpha }$, a Markovian kernel on E, as follows:*

(4) $$K_{\alpha }\left(x,A\right):=K\left(x,A\right)+\left(1-K(x,E)\right)\alpha \left(A\right)$$

*for all x∈E and A∈B(E).*

$K_{\alpha }$ is a Markovian kernel because $K_{\alpha }\left(x,E\right)=1$. It is easy to sample $X_{t+1}∼K_{\alpha }\left(X_{t},\cdot \right)$ from any state $X_{t}\in E$: run the transition as normal by using $\tilde{K}$ to have a next state denoted by *Y*, then $X_{t+1}=Y$ if Y∈E; otherwise, sample $X_{t+1}$ from α.

We know that α is the quasi-stationary distribution of *K* if and only if it is the stationary distribution of $K_{\alpha }$.

(5) $$\alpha =\alpha K_{\alpha }.$$

It is easy to observe that α=β if and only if $K_{\alpha }=K_{\beta }$ for any two distributions α and β. Moreover, for every $\alpha^{\prime }$, $K_{\alpha^{\prime }}$ has a unique invariant probability denoted by $\Gamma (\alpha^{\prime })$. Then, $\alpha^{\prime }\mapsto \Gamma \left(\alpha^{\prime }\right)$ is continuous in P(E) (i.e., for the topology of weak convergence), and there exists α∈P(E) such that α=Γ(α) or, equivalently, α is a QSD for *K*.

### 2.2. Review of Simulation Methods for Quasi-Stationary Distribution

According to the above subsection, the QSD α satisfies the fixed point problem as follows:

(6) α=Γ(α),

where Γ(α) is the stationary distribution of $K_{\alpha }$ on E. In general, (6) can be solved recursively by $\alpha_{n+1}\leftarrow \Gamma \left(\alpha_{n}\right)$.

The Fleming–Viot (FV) method [19] evolves *N* particles independently of each other as a Markov process associated with the transition kernel $K_{\alpha }$ until one succeeds in jumping to the absorbing state *∂*. At that time, this killed particle is immediately reset to E as an initial state uniformly chosen from one of the remaining N−1 particles. The QSD α is approximated by the empirical distribution of the *N* particles in total, and these particles can be regarded as samples from the quasi-stationary distribution α such as the MCMC method.

Ref. [37] proposed a simulation method by only using one particle at each iteration to update α. At iteration *n*, given an $\alpha_{n}\in P\left(E\right)$, one can run a discrete-time Markov chain $X^{\left(n+1\right)}$ as normal on ∂∪E with initial $X_{0}^{\left(n+1\right)}∼\alpha_{n}$; then, $\alpha_{n+1}$ is computed as the following weighted average of empirical distributions:

(7) $$\begin{array}{c} \alpha_{n+1}\left(x\right):=\alpha_{n}\left(x\right)+\frac{1}{n+1}\sum_{k=0}^{\tau^{\left(n+1\right)}-1}\frac{I\left(X_{k}^{\left(n+1\right)}=x|X_{0}^{\left(n+1\right)}∼\alpha_{n}\right)-\alpha_{n}\left(x\right)}{\frac{1}{n+1}\sum_{j=1}^{n+1}\tau^{\left(j\right)}} \end{array}$$

where n≥0 and *I* are the indicator functions, and $\tau^{\left(j\right)}=\min\left\{k\geq 0|X_{k}^{\left(j\right)}\in \partial \right\}$ is the first extinction time for the process $X^{\left(j\right)}$. This iterative scheme has a convergence rate of $O(\frac{1}{\sqrt{n}})$.

In [23,24], the above method is extended to the stochastic approximations framework:

(8) $$\alpha_{n+1}\left(x\right)=\Theta_{H}\left[\alpha_{n}+\epsilon_{n}\sum_{k=0}^{\tau^{\left(n+1\right)}-1}\left(I\left(X_{k}^{\left(n+1\right)}=x|X_{0}^{\left(n+1\right)}∼\alpha_{n}\right)-\alpha_{n}\left(x\right)\right)\right]$$

where $\Theta_{H}$ denotes the $L_{2}$ projection into the probability simplex, and $\epsilon_{n}$ is the step size satisfying $\sum \epsilon_{n}=\infty$ and $\sum \epsilon_{n}^{2}<\infty$. Specifically, if $\epsilon_{n}=O\left(\frac{1}{n^{r}}\right)$ for 0.5<r<1, under a sufficient condition, they have $\sqrt{n^{r}}\left(\alpha_{n}-\alpha \right)\overset{d}{\to }N\left(0,V\right)$ for some matrix *V* [23,24]. If the Polyak–Ruppert averaging technique is applied to generate the following:

(9) $$\nu_{n}:=\frac{1}{n}\sum_{k=1}^{n}\alpha_{k},$$

then the convergence rate of $\nu_{n}\to \alpha$ becomes $\frac{1}{\sqrt{n}}$ [23,24].

The simulation schemes (7) and (8) need to sample the initial states according to $\alpha_{n}$ and to add the empirical distribution and $\alpha_{n}$ at each *x* point wisely. Thus, they are suitable for finite state space where α is a probability vector saved in the tabular form. In (8), there is no need to record all exit times $\tau^{\left(j\right)},j=1,\ldots ,n$, but the additional projection operation in (8) is computationally expensive since the cost is O(mlogm) where m=|E| [38,39].

## 3. Learn Quasi-Stationary Distribution

We focus on the computation of the expression of the quasi-stationary distribution. In particular, when this distribution is parametrized in a certain manner by θ, we can extend the tabular form for finite-state Markov chain to any flexible form, even in the neural networks for probability density function in $R^{d}$. However, we do not pursue this representation and expressivity issue here and restrict our discussion to finite state space only to illustrate our main idea first. In finite state space, α(x) for x∈E={1,…,m} can be simply described as a softmax function with m−1 parameter $\theta_{i}:\alpha \left(i\right)\propto e^{\theta_{i}},1\leq i\leq m-1$ ($\theta_{m}=0$). This introduces no representation error. For the generalization to continuous space E in jump and diffusion processes or even for a huge finite state space, a good representation of $\alpha_{\theta }\left(x\right)$ is important in practice.

In this section, we shall formulate our QSD problem in terms of reinforcement learning (RL) so that the problem of seeking optimal parameters becomes a policy optimization problem. We derive the policy gradient theorem to construct a gradient descent method for the optimal parameter. We then show a method for designing actor-critic algorithms based on stochastic optimization.

### 3.1. Formulation of RL and Policy Gradient Theorem

Before introducing the RL method of our QSD problem, we develop a general formulation by introducing the KL-divergence between two path distributions.

Let $P_{\theta }$ and $Q_{\theta }$ be two families of Markovian kernels on E in parametric forms with the same set of parameters θ∈Θ. Assume both $P_{\theta }$ and $Q_{\theta }$ are ergodic for any θ. Let T>0 and denote a path up to time *T* by $\omega_{0}^{T}=\left(X_{0},X_{1},\ldots ,X_{T}\right)\in E^{T+1}$. Define the path distributions under the Markov chain kernel $P_{\theta }$ and $Q_{\theta }$, respectively.

(10) $$P_{\theta }\left(\omega_{0}^{T}\right):=\prod_{t=1}^{T}P_{\theta }\left(X_{t}|X_{t-1}\right),\;Q_{\theta }\left(\omega_{0}^{T}\right):=\prod_{t=1}^{T}Q_{\theta }\left(X_{t}|X_{t-1}\right).$$

Define the KL divergence from $P_{\theta }$ to $Q_{\theta }$ on $E^{T+1}$:

(11) $$D_{KL}\left(P_{\theta }|Q_{\theta }\right):=\sum_{\omega_{0}^{T}}P_{\theta }\left(\omega_{0}^{T}\right)\ln\frac{P_{\theta }\left(\omega_{0}^{T}\right)}{Q_{\theta }\left(\omega_{0}^{T}\right)}=-E_{P_{\theta }}\sum_{t=1}^{T}R_{\theta }\left(X_{t-1},X_{t}\right),$$

where the expectation $E_{P_{\theta }}$ is for the path $\left(X_{0},X_{1},\ldots ,X_{T}\right)$ generated by the transition kernel $P_{\theta }$, and the following is called the (one-step) **reward**.

(12) $$R_{\theta }\left(X_{t-1},X_{t}\right):=-\ln\frac{P_{\theta }\left(X_{t}|X_{t-1}\right)}{Q_{\theta }\left(X_{t}|X_{t-1}\right)}.$$

Define the **average reward** r(θ) as the time averaged negative KL divergence in the limit of T→∞.

(13) $$\begin{array}{cc} r(\theta ) & :=-\lim_{T\to \infty }\frac{1}{T}D_{KL}\left(P_{\theta }|Q_{\theta }\right)=-\lim_{T\to \infty }\frac{1}{T}E_{P_{\theta }}\sum_{t=1}^{T}R_{\theta }\left(X_{t-1},X_{t}\right). \end{array}$$

Due to ergodicity of $P_{\theta }$, $r\left(\theta \right)=\sum_{x_{0},x_{1}}R_{\theta }\left(x_{0},x_{1}\right)P_{\theta }\left(x_{1}|x_{0}\right)\mu_{\theta }\left(x_{0}\right)$ where $\mu_{\theta }$ is the invariant measure of $P_{\theta }$, r(θ) is independent of initial state $X_{0}$. Obviously, r(θ)≤0 for any θ.

**Property** **1.** *The following are equivalent:*

*1.*  *r(θ) reaches its maximal value* 0 at $\theta^{*}$;
*2.*  $P_{\theta^{*}}=Q_{\theta^{*}}$ in $P(E^{T+1})$ for any T>0;
*3.*  $P_{\theta^{*}}=Q_{\theta^{*}}$;
*4.*  $R_{\theta^{*}}\equiv 0$.

**Proof.**  We only need to show (1)⟹(3). It is easy to see that

$$r\left(\theta \right)=-\sum_{x_{0}}\;D_{KL}\left(P_{\theta }\left(\cdot |x_{0}\right)\;|\;Q_{\theta }\left(\cdot |x_{0}\right)\right)\;\mu_{\theta }\left(x_{0}\right).$$

If r(θ)=0, since $\mu_{\theta }>0$, then

$$D_{KL}\left(P_{\theta }\left(\cdot |x_{0}\right)\;|\;Q_{\theta }\left(\cdot |x_{0}\right)\right)=0\;\;\;\forall x_{0}.$$

Thus, we have $P_{\theta }=Q_{\theta }$. □

The above property establishes the relationship between the RL problem and QSD problem.

We show our theoretic main result below as the foundation of our algorithm to be developed later. This theorem can be regarded as one type of the policy gradient theorem for the policy gradient method in reinforcement learning [31].

Define the following **value function** ([31] Chapter 13).

(14) $$V\left(x\right):=\lim_{T\to \infty }\sum_{t=1}^{T}E_{P_{\theta }}\left[R_{\theta }\left(X_{t-1},X_{t}\right)-r\left(\theta \right)|X_{0}=x\right].$$

Certainly, *V* also depends on θ, although we do not write θ explicitly.

**Theorem** **1(policy gradient theorem).**  *We have the following two properties:*

*1.*  *At any θ, for any x∈E, the following Bellman-type equation holds for the value function V and the average reward r(θ):*
(15) $$V\left(x\right)=E_{Y∼P_{\theta }\left(\cdot |x\right)}\left[V\left(Y\right)+R_{\theta }\left(x,Y\right)-r\left(\theta \right)\right].$$
*2.*  *The gradient of the average reward r(θ) is the following:*
(16) $$\begin{array}{cc} \nabla_{\theta }r\left(\theta \right) & =E\left[\nabla_{\theta }\ln Q_{\theta }\left(Y|X\right)\right]+\\ \; & \;E\left[\left(V\left(Y\right)-V\left(X\right)+R_{\theta }\left(X,Y\right)-r\left(\theta \right)\right)\nabla_{\theta }\ln P_{\theta }\left(Y|X\right)\right], \end{array}$$

*where expectations are for the joint distribution $\left(X,Y\right)∼\mu_{\theta }\left(x\right)P_{\theta }\left(y|x\right)$ where $\mu_{\theta }$ is the stationary measure of $P_{\theta }$.*

**Proof.**  We shall prove the Bellman equation first and then we use the Bellman equation to derive the gradient of the average reward r(θ). For any $x_{0}\in E$, by writing $\omega_{0}^{T}=\left(x_{0},\ldots ,x_{T}\right)$ and defining

$$\Delta R_{\theta }\left(\omega_{0}^{T}\right)=\sum_{t=1}^{T}\left(R\left(x_{t-1},x_{t}\right)-r\left(\theta \right)\right),$$

we have the following:

(17) $$\begin{array}{cc} V\left(x_{0}\right) & =\lim_{T\to \infty }E_{P_{\theta }}\left[\Delta R_{\theta }\left(\omega_{0}^{T}\right)|X_{0}=x\right]\\ \; & =\lim_{T\to \infty }\sum_{x_{2},\ldots ,x_{T}}\sum_{x_{1}}\left(\left(\prod_{t=2}^{T}P_{\theta }\left(x_{t}|x_{t-1}\right)\right)P_{\theta }\left(x_{1}|x_{0}\right)\Delta R\left(\omega_{0}^{T}\right)\right)\\ \; & =\lim_{T\to \infty }\sum_{x_{1}}\left(P_{\theta }\left(x_{1}|x_{0}\right)\sum_{x_{2},\ldots ,x_{T}}\left(\prod_{t=2}^{T}P_{\theta }\left(x_{t}|x_{t-1}\right)\left[\Delta R\left(\omega_{1}^{T}\right)+\Delta R\left(\omega_{0}^{1}\right)\right]\right)\right)\\ \; & =\sum_{x_{1}}\left(P_{\theta }\left(x_{1}|x_{0}\right)\left(\lim_{T\to \infty }\left[\sum_{x_{2},\ldots ,x_{T}}\prod_{t=2}^{T}P_{\theta }\left(x_{t}|x_{t-1}\right)\Delta R\left(\omega_{1}^{T}\right)\right]+\Delta R\left(\omega_{0}^{1}\right)\right)\right)\\ \; & =\sum_{x_{1}}P_{\theta }\left(x_{1}|x_{0}\right)\left[V\left(x_{1}\right)+R_{\theta }\left(x_{0},x_{1}\right)\right]-r\left(\theta \right), \end{array}$$

which proves (15); in other words, we have the following.

$$r\left(\theta \right)=E_{Y∼P_{\theta }\left(\cdot |x\right)}\left[V\left(Y\right)+R_{\theta }\left(x,Y\right)-V\left(x\right)\right],\;\forall x\in E.$$

Next, we compute the gradient of r(θ). By trivial equality of the following:

(18) $$\sum_{x_{1}}P_{\theta }\left(x_{1}|x_{0}\right)\nabla_{\theta }\ln P_{\theta }\left(x_{1}|x_{0}\right)=\nabla_{\theta }\sum_{x_{1}}P_{\theta }\left(x_{1}|x_{0}\right)=0,$$

and the definition (12), we can write the gradient of r(θ) as follows.

$$\begin{array}{cc} \nabla_{\theta }r\left(\theta \right)= & \sum_{y}\nabla_{\theta }P_{\theta }\left(y|x\right)\left[V\left(y\right)+R_{\theta }\left(x,y\right)-V\left(x\right)\right]\\ \; & +\sum_{y}P_{\theta }\left(y|x\right)\left[\nabla_{\theta }V\left(y\right)-\nabla_{\theta }V\left(x\right)+\nabla_{\theta }\ln Q_{\theta }\left(y|x\right)\right]. \end{array}$$

We here keep the term V(x) in the first line, even though it has no contribution here (in fact, to add any constant to V(x) is also fine). Since this equation holds for all states *x* on the right-hand side, we take the expectation with respect to $\mu_{\theta }$, the stationary distribution of $P_{\theta }$. Thus, we have the following.

$$\begin{array}{cc} \nabla_{\theta }r\left(\theta \right)= & \sum_{x,y}\mu_{\theta }\left(x\right)\nabla_{\theta }P_{\theta }\left(y|x\right)\left[V\left(y\right)+R_{\theta }\left(x,y\right)-V\left(x\right)\right]\\ \; & +\sum_{x,y}\mu_{\theta }\left(x\right)P_{\theta }\left(y|x\right)\left[\nabla_{\theta }V\left(y\right)-\nabla_{\theta }V\left(x\right)+\nabla_{\theta }\ln Q_{\theta }\left(y|x\right)\right]\\ = & \sum_{x,y}\mu_{\theta }\left(x\right)\nabla_{\theta }P_{\theta }\left(y|x\right)\left[V\left(y\right)+R_{\theta }\left(x,y\right)-V\left(x\right)\right]\\ \; & +\sum_{y}\mu_{\theta }\left(y\right)\nabla_{\theta }V\left(y\right)-\sum_{x}\mu_{\theta }\left(x\right)\nabla_{\theta }V\left(x\right)+\sum_{x,y}\mu_{\theta }\left(x\right)P_{\theta }\left(y|x\right)\nabla_{\theta }\ln Q_{\theta }\left(y|x\right)\\ = & \sum_{x,y}\mu_{\theta }\left(x\right)P_{\theta }\left(y|x\right)\left[V\left(y\right)+R_{\theta }\left(x,y\right)-V\left(x\right)\right]\;\nabla_{\theta }\ln P_{\theta }\left(y|x\right)\\ \; & +\sum_{x,y}\mu_{\theta }\left(x\right)P_{\theta }\left(y|x\right)\nabla_{\theta }\ln Q_{\theta }\left(y|x\right). \end{array}$$

In fact, we can add any constant number *b* (independent of *x* and *y*) inside the squared bracket of the last line without changing the equality due to the following fact similar to (18): $\sum_{x,y}\mu_{\theta }\left(x\right)\nabla_{\theta }P_{\theta }\left(y|x\right)=\sum_{y}\mu_{\theta }\left(y\right)\nabla_{\theta }\sum_{x}P_{\theta }\left(x|y\right)=0$. (16) is a special case of b=r(θ). □

**Remark** **1.** *As shown in the proof, (16) holds if r(θ) at the right-hand side is replaced by any constant number b. b=r(θ) is a good choice to reduce the variance since r(θ) can be regarded as the expectation of $R_{\theta }$.*

**Remark** **2.** *If $P_{\theta }=Q_{\theta }$, then the first term of (16) vanishes due to (18) and the second term of (16) vanishes due to (15).*

**Remark** **3.** *The name of “policy” here refers to the role of θ as the policy for decision makers to improve reward r(θ).*

### 3.2. Learn QSD

Now, we discuss how to connect QSD with the results in the previous subsection. In view of Equation (5), we introduce $\beta :=\alpha K_{\alpha }$ as the one-step distribution if starting from the initial α; in other words, we have the following.

(19) $$\beta \left(y\right):=\sum_{x\in E}\alpha \left(x\right)K_{\alpha }\left(x,y\right),\;\;\;\forall y$$

By (5), α is a QSD if and only if β=α. However, we do not directly compare these two distributions α and β. Instead, we consider their Markovian kernels induced by (4): $K_{\alpha }$ and $K_{\beta }$. Our approach is to consider KL divergence similar to (11) between two kernels $K_{\alpha }$ and $K_{\beta }$ since α=β if and only if $K_{\alpha }=K_{\beta }$. In this manner, one can view $K_{\alpha }$ and $K_{\beta }$ (note $\beta =\alpha K_{\alpha }$) as two transition matrices $P_{\theta }$ and $Q_{\theta }$ in the previous section, in which the parameter θ here is in fact the distribution α.

To have a further representation of the distribution α, which is a (probability mass) function on E, we propose a parametrized family for α in the form $\alpha_{\theta }$ where θ is a generic parameter. In the simplest case, $\alpha_{\theta }$ takes the so-called *soft-max* form $\alpha_{\theta }\left(i\right)=\frac{e^{\theta_{i}}}{\sum_{j\geq 1}e^{\theta_{j}}}$ if E={1,…,N} for $\theta =(\theta_{1},\ldots ,\theta_{N-1},\theta_{N}\equiv 0).$ This parametrization represents α without any approximation error for finite state space and the effective space of θ is just $R^{N-1}$. For certain problems, particularly with large state space, if one has some prior knowledge about the structure of the function α on E, one might propose other parametric forms of $\alpha_{\theta }$ with the dimension of θ less than the cardinality |E| to improve the efficiency, although the extra representation error in this manner has to be introduced.

For any given $\alpha_{\theta }\in P\left(E\right)$, the corresponding Markovian kernel $K_{\alpha_{\theta }}$ is then defined in (4) and $\beta_{\theta }=\alpha_{\theta }K_{\alpha_{\theta }}i$ is defined by (19). $K_{\beta_{\theta }}$ is like-wise defined by (4) again. To use the formulation in Section 3.1, we chose $P_{\theta }=K_{\alpha_{\theta }}$ and $Q_{\theta }=K_{\beta_{\theta }}$. Define the objective function as before:

$$\begin{array}{cc} r(\theta ) & :=-\lim_{T\to \infty }\frac{1}{T}D_{KL}\left(P_{\theta }|Q_{\theta }\right)=-\lim_{T\to \infty }\frac{1}{T}E_{P_{\theta }}\sum_{t=1}^{T}R_{\theta }\left(X_{t-1},X_{t}\right). \end{array}$$

where the following is the case.

$$R_{\theta }\left(x,y\right)=-\ln\frac{K_{\alpha_{\theta }}\left(x,y\right)}{K_{\beta_{\theta }}\left(x,y\right)}.$$

The value function V(x) is defined similarly. Theorem 1 now provides the expression of the following gradient:

(20) $$\begin{array}{cc} \nabla_{\theta }r\left(\theta \right) & =E[\left(R_{\theta }\left(X,Y\right)-r\left(\theta \right)+V\left(Y\right)-V\left(X\right)\right)\nabla_{\theta }\ln K_{\alpha_{\theta }}\left(X,Y\right)\\ & \;+\nabla_{\theta }\ln K_{\beta_{\theta }}\left(X,Y\right)] \end{array}$$

where $\left(X,Y\right)∼\mu_{\theta }\left(x\right)K_{\alpha_{\theta }}\left(x,y\right)$ and where $\mu_{\theta }$ is the stationary measure of $K_{\alpha_{\theta }}$.

The optimal $\theta^{*}$ for the QSD $\alpha_{\theta }$ is to maximize r(θ), and this can be solved by the gradient descent algorithm:

(21) $$\theta_{t+1}=\theta_{t}+\eta_{t}^{\theta }\nabla_{\theta }r\left(\theta_{t}\right).$$

where $\eta_{t}^{\theta }>0$ is the step size. In practice, the stochastic gradient is applied:

$$\nabla_{\theta }r\left(\theta_{t}\right)\approx \nabla_{\theta }\ln K_{\alpha_{\theta }}\left(X_{t},X_{t+1}\right)\times \delta \left(X_{t},X_{t+1}\right)+\nabla_{\theta }\ln K_{\beta_{\theta }}\left(X_{t},X_{t+1}\right)$$

where $X_{t},X_{t+1}$ are sampled based on the Markovian kernel $K_{\alpha_{\theta }}$ (see Algorithm 1) and the differential temporal (TD) error $\delta_{t}$ is as follows.

(22) $$\delta_{t}=\delta \left(X_{t},X_{t+1}\right)=R_{\theta }\left(X_{t},X_{t+1}\right)-r\left(\theta_{t}\right)+V\left(X_{t+1}\right)-V\left(X_{t}\right).$$

Next, we need to address a remaining issue, which is the question of how to compute value functions *V* and $r(\theta_{t})$ in the TD error (22). In addition, we also need to show the details of computing $\nabla_{\theta }K_{\alpha_{\theta }}$ and $\nabla_{\theta }K_{\beta_{\theta }}$.

### 3.3. Actor-Critic Algorithm

With the stochastic gradient method (21), we can obtain optimal policy $\theta^{*}$. We refer to (21) as the learning dynamics for the policy, and it is generally known as *actor*. To calculate the value function *V* appearing in ∇r(θ), we need to have a new learning dynamic, which is called *critic*. Then, the overall policy-gradient method is termed as the actor-critic method.

We start with the Bellman Equation (15) for the value function and considered the mean-square-error loss as follows:

$$\mathrm{MSE}\left[V\right]=\frac{1}{2}\sum_{x}\nu \left(x\right){\left(\sum_{y}K_{\alpha_{\theta }}\left(x,y\right)\left[V\left(y\right)+R_{\theta }\left(x,y\right)-r\left(\theta \right)\right]-V\left(x\right)\right)}^{2}$$

where ν is any distribution supported on E. MSE[V]=0 if and only if *V* satisfies the Bellman Equation (15), i.e., *V* is the value function. To learn *V*, we introduce function approximation for the value function, $V_{\psi }$, with the parameter ψ and considered to minimize the following:

$$\mathrm{MSE}\left(\psi \right)=\frac{1}{2}\sum_{x}\nu \left(x\right){\left(\sum_{y}K_{\alpha_{\theta }}\left(x,y\right)\left[V\left(y\right)+R_{\theta }\left(x,y\right)-r\left(\theta \right)\right]-V_{\psi }\left(x\right)\right)}^{2}$$

by the semi-gradient method ([31], Chapter 9).

$$\begin{array}{cc} \nabla_{\psi }\mathrm{MSE}\left(\psi \right) & =-\sum_{x,y}\nu \left(x\right)K_{\alpha_{\theta }}\left(x,y\right)\left[V\left(y\right)+R_{\theta }\left(x,y\right)-r\left(\theta \right)-V_{\psi }\left(x\right)\right]\nabla_{\psi }V_{\psi }\left(x\right)\\ \; & \approx -\sum_{x,y}\nu \left(x\right)K_{\alpha_{\theta }}\left(x,y\right)\left[V_{\psi }\left(y\right)+R_{\theta }\left(x,y\right)-r\left(\theta \right)-V_{\psi }\left(x\right)\right]\nabla_{\psi }V_{\psi }\left(x\right) \end{array}$$

Here, the term V(y) is frozen first and then approximated by $V_{\psi }$ since it could be treated as a prior guess of the value function for the future state.

Then, for the gradient descent iteration $\psi_{t+1}=\psi_{t}-\eta_{t}^{\psi }\nabla_{\psi }{\mathrm{MSE}}_{V}\left(\psi_{t}\right)$ where $\eta_{t}^{\psi }$ is the step size, we can have the following stochastic gradient iteration:

(23) $$\psi_{t+1}=\psi_{t}+\eta_{t}^{\psi }\;\delta \left(X_{t},X_{t+1}\right)\;\nabla_{\psi }V_{\psi_{t}}\left(X_{t}\right)$$

where the differential temporal (TD) error δ is defined above in (22).

$$\delta_{t}=\delta \left(X_{t},X_{t+1}\right)=R_{\theta_{t}}\left(X_{t},X_{t+1}\right)-r\left(\theta_{t}\right)+V_{\psi_{t}}\left(X_{t+1}\right)-V_{\psi_{t}}\left(X_{t}\right).$$

Here, for the sake of simplicity, $\left(X_{t},X_{t+1}\right)$ are the same samples as in the actor method for $\theta_{t}$. This means that distribution ν above is chosen as μ used for the gradient $\nabla_{\theta }r\left(\theta \right)$.

Next, we consider the calculation of the reward r(θ) by the following Bellman Equation (15).

$$\sum_{x}\mu \left(x\right)\sum_{y}K_{\alpha_{\theta }}\left(x,y\right)\left(R_{\theta }\left(x,y\right)-r\left(\theta \right)+V\left(y\right)-V\left(x\right)\right)=0$$

Let $r_{t}$ be the estimate of the reward $r(\theta_{t})$ at time *t*. We can update our estimate of the reward every time a transition occurs as follows:

(24) $$r_{t+1}=r_{t}+\eta_{t}^{r}\times \delta_{t}$$

where $\delta_{t}$ is the TD error before

$$\delta_{t}=\delta \left(X_{t},X_{t+1}\right)=R_{\theta_{t}}\left(X_{t},X_{t+1}\right)-r_{t}+V_{\psi_{t}}\left(X_{t+1}\right)-V_{\psi_{t}}\left(X_{t}\right).$$

In conclusion, (21), (23) and (24) together consist of the actor-critic algorithm, which is summarized in Algorithm 1. We remark that Algorithm 1 can be easily adapted to use the mini-batch gradient method where several copies of $\left(X_{t},X_{t+1}\right)$ are sampled, and the average is used to update the parameters. The stationary distribution $\mu_{\theta }$ of $K_{\alpha_{\theta }}$ is sampled by running the corresponding Markov chain for several steps with “warm start”: the initial for $\theta_{t+1}$ is set as the final state generated from the previous iteration at $\theta_{t}$. The length of this “burn-in” period can be set as just one step in practice for efficiency.

**Remark** **4.** *Finally, we remark on the computation of $\nabla_{\theta }\ln K_{\alpha_{\theta }}$ and $\nabla_{\theta }\ln K_{\beta_{\theta }}$ in Algorithm 1. The details are shown in Appendix A. We comment that the main computational cost is the function K(x,E), which has to be pre-computed and stored. If the problem has some special structure, the function could be approximated in parametric form. Another special case is our second example where K(x,E)=0∀x∈{2,3,…,N}.*

## 4. Numerical Experiment

In this section, we present two examples to demonstrate Algorithm 1. We call the algorithm (7), (8) and (9) in Section 2.2 used in [23,24], as **Vanilla Algorithm**, **Projection Algorithm** and **Polyak Averaging Algorithm**, respectively. Let 0 be the absorbing state and E={1,…,N} are non-absorbing states; the Markov transition matrix on {0,…,N} is denoted by the following:

$$\tilde{K}=\left[\begin{array}{cc} 1 & 0\\ {}* & K \end{array}\right],$$

where *K* is an *N*-by-*N* sub-Markovian matrix. For Algorithm 1, distribution $\alpha_{\theta }$ on E is always parameterized as follows:

$$\alpha_{\theta }=\frac{1}{e^{\theta_{1}}+\ldots +e^{\theta_{N-1}}+1}\left[e^{\theta_{1}},\ldots ,e^{\theta_{N-1}},1\right],$$

and the value function $V_{\psi }\left(x\right)$ is represented in tabular form for simplicity:

$$V_{\psi }=\left[\psi_{1},\ldots ,\psi_{N}\right]$$

where $\psi \in R^{N}$.

### 4.1. Loopy Markov Chain

We test a toy example of the three-state loopy Markov chain, which was considered in [23,24]. The transition probability matrix for the four states {0,1,2,3} is as follows.

$$\tilde{K}=\left[\begin{array}{cccc} 1 & 0 & 0 & 0\\ \epsilon & \frac{1-\epsilon }{3} & \frac{1-\epsilon }{3} & \frac{1-\epsilon }{3}\\ \epsilon & \frac{1-\epsilon }{3} & \frac{1-\epsilon }{3} & \frac{1-\epsilon }{3}\\ \epsilon & \frac{1-\epsilon }{3} & \frac{1-\epsilon }{3} & \frac{1-\epsilon }{3} \end{array}\right],\;\epsilon \in \left(0,1\right).$$

The state 0 is the absorbing state *∂* and E={1,2,3}. *K* is the sub-matrix of $\tilde{K}$ corresponding to the states {1,2,3}. With the probability ϵ, the process exits E directly from state 1, 2 or 3. The true quasi-stationary distribution of this example is the uniform distribution for any ϵ.

In order to show the advantage of our algorithm, we consider two cases: (1) ϵ=0.1 and (2) ϵ=0.9. For a larger ϵ, the original Markov chain is very easy to exit; thus, each iteration takes less time, but the convergence rate of Vanilla algorithm is slower.

In order to quantify the accuracy of the learned quasi-stationary distribution, we compute the $L_{2}$ norm of the error between the learned quasi-stationary distribution and the true values.

**Figure 1:**
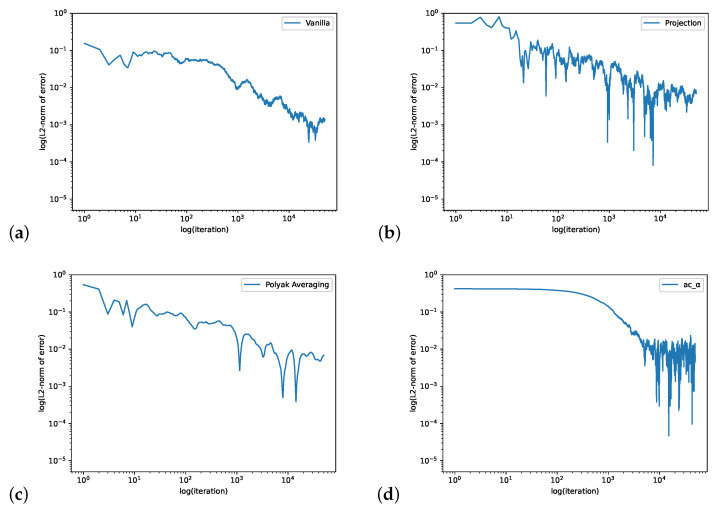
The loopy Markov chain example with ϵ=0.1. The figure shows the log–log plots of $L_{2}$-norm error of the Vanilla Algorithm (**a**), Projection Algorithm (**b**), Polyak Averaging Algorithm (**c**) and our actor-critic algorithm (**d**). The iteration for the actor-critic algorithm is defined as one step of gradient descent (“*t*” in Algorithm 1).

**Figure 2:**
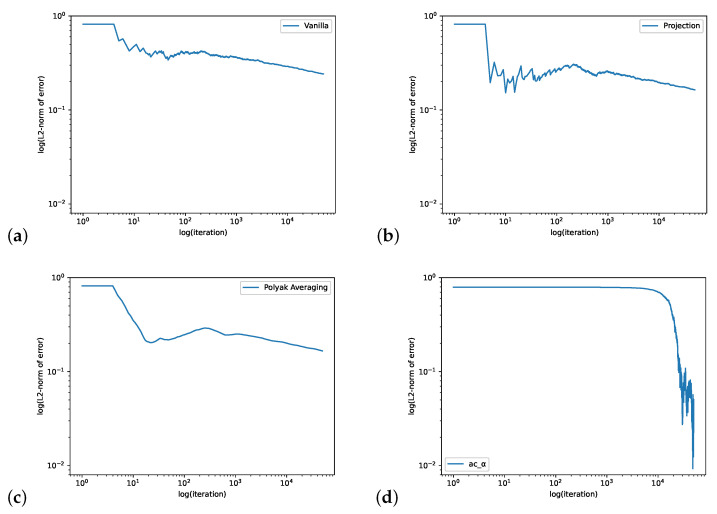
The loopy Markov chain example with ϵ=0.9. The figure shows the log–log plots of $L_{2}$-norm error of Vanilla Algorithm (**a**), Projection Algorithm (**b**), Polyak Averaging Algorithm (**c**) and our actor-critic algorithm (**d**).

In Figure 1, we compute the QSD when ϵ=0.1. We set the initial value $\theta_{0}=\left[-1,1\right],\psi_{0}=\left[0,0,0\right],r_{0}=0$, the learning rate $\eta_{n}^{\theta }=\max\left\{1/n^{0.1},0.2\right\},\eta_{n}^{\psi }=0.0001,\eta_{n}^{r}=0.0001$ and the batch size is 4. The step size for the Projection Algorithm is $\epsilon_{n}=n^{-0.99}$. Figure 2 is for the case when ϵ=0.9 We set the initial value $\theta_{0}=\left[4,-2\right],\psi_{0}=\left[0,0,0\right],r_{0}=0$, the learning rate $\eta_{n}^{\theta }=0.04,\eta_{n}^{\psi }=0.0001,\eta_{n}^{r}=0.0001$ and the batch size is 32. The step size for the Projection Algorithm is $\epsilon_{n}=n^{-0.99}$.

### 4.2. M/M/1/N Queue with Finite Capacity and Absorption

Our second example is an M/M/1 queue with finite queue capacity. The 0 state has been set as an absorbing state. The transition probability matrix on {0,…,N} takes the following form:

$$\tilde{K}=\left[\begin{array}{cccccccc} 1 & 0 & 0 & 0 & 0 & \ldots & 0 & 0\\ \mu_{1} & 0 & \lambda_{1} & 0 & 0 & \ldots & 0 & 0\\ 0 & \mu_{2} & 0 & \lambda_{2} & 0 & \ldots & 0 & 0\\ 0 & 0 & \mu_{3} & 0 & \lambda_{3} & \ldots & 0 & 0\\ \vdots & \vdots & \vdots & \vdots & \vdots & & \vdots & \vdots \\ 0 & 0 & 0 & 0 & 0 & & 0 & \lambda_{N-1}\\ 0 & 0 & 0 & 0 & 0 & \ldots & 1 & 0 \end{array}\right]$$

where $\lambda_{i}=\frac{\rho_{i}}{\rho_{i}+1}$, $\mu_{i}=\frac{1}{\rho_{i}+1}$, i∈{1,2,…,N−1}. $\rho_{i}>1$ means a higher chance to jump to the right than to the left. A larger $\rho_{i}$ will have less probability of exiting E. Note that K(x,E)=1 for x∈{2,…,N}. Thus, $K_{\alpha }\left(x,y\right)=K\left(x,y\right)$ for any α if x≠1 and $K_{\alpha }\left(1,y\right)=K\left(1,y\right)+\mu_{1}\alpha \left(y\right)=\left\{\begin{array}{cc} \lambda_{1}+\mu_{1}\alpha \left(1\right) & y=1,\\ \mu_{1}\alpha \left(y\right) & 2\leq y\leq N \end{array}\right..$ Then, $R_{\theta }\left(x,y\right)=-\ln\frac{K_{\alpha_{\theta }}\left(x,y\right)}{K_{\beta_{\theta }}\left(x,y\right)}=0$ if x≠1 and by (20), the gradient is simplified as follows:

$$\nabla_{\theta }r\left(\theta \right)=E_{Y}\left[\left(R_{\theta }\left(1,Y\right)-r\left(\theta \right)+V\left(Y\right)-V\left(1\right)\right)\nabla_{\theta }\ln K_{\alpha_{\theta }}\left(1,Y\right)+\nabla_{\theta }\ln K_{\beta_{\theta }}\left(1,Y\right)\right]$$

where *Y* follows distribution $K_{\alpha }\left(1,\cdot \right)$.

**Figure 3:**
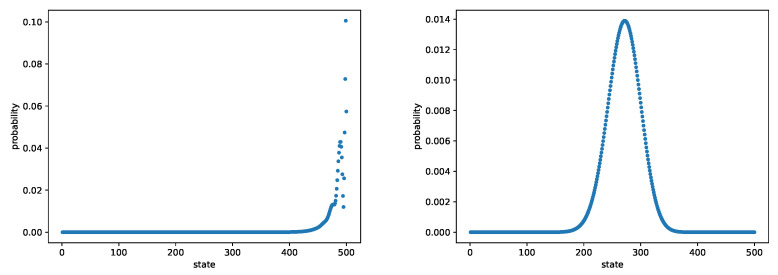
The QSD for M/M/1/500 queue with $\rho_{i}\equiv 1.25$ (**left**) and $\rho_{i}=2-\frac{3}{2N-4}\left(i-1\right)$ (**right**).

We consider two cases: (1) a constant $\rho_{i}=1.25$ and (2) a state-dependent $\rho_{i}=2-\frac{3}{2N-4}\left(i-1\right)$. Note that $\rho_{i}=1$ gives an equal probability of jumping to the left and to the right. Thus, in case (1), there is a boundary layer at the most right end and in case (2), we expect to see a peak of the QSD near i≈2N/3. Figure 3 shows the true QSD in both cases. We set N=500.

**Figure 4:**
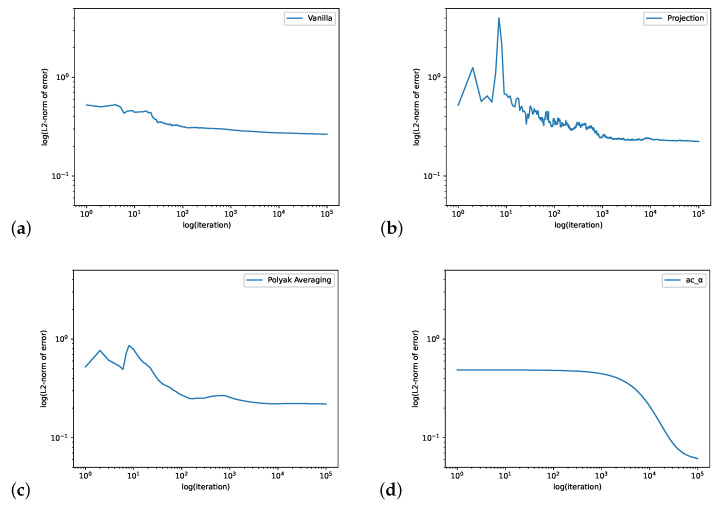
The M/M/1/500 queue with $\rho_{i}=1.25$. The figure shows the log–log plots of $L_{2}$-norm error of Vanilla Algorithm (**a**), Projection Algorithm (**b**), Polyak Averaging Algorithm (**c**) and our actor-critic algorithm (**d**).

**Figure 5:**
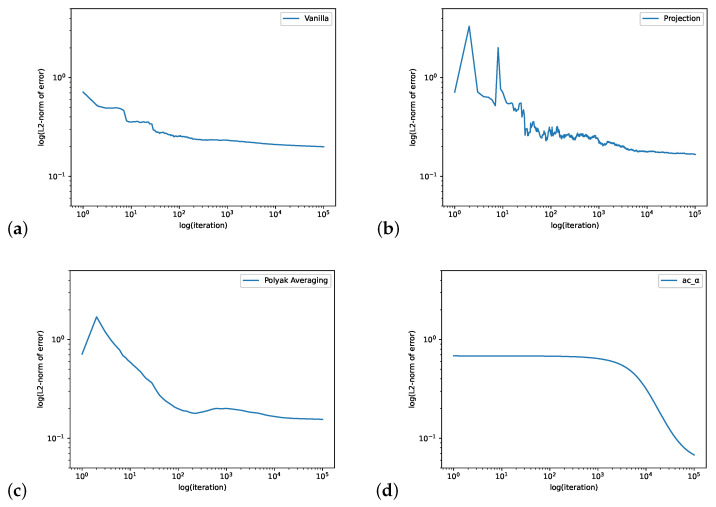
The M/M/1/500 queue with $\rho_{i}=2-\frac{3}{2N-4}\left(i-1\right)$. The figure shows the log–log plots of $L_{2}$-norm error of Vanilla Algorithm (**a**), Projection Algorithm (**b**), Polyak Averaging Algorithm (**c**) and our actor-critic algorithm (**d**).

In Figure 4, we consider the case when $\rho_{i}=1.25$ and compute $L_{2}$ errors. We set the initial value $\theta_{0}^{i}=-35+\frac{35}{498}\left(i-1\right)$ for i∈{1,2,…,498} and $\theta_{0}^{499}=3$, $\psi_{0}=\left[0,0,\ldots ,0\right]$, $r_{0}=0$ and the learning rate $\eta_{n}^{\theta }=0.0003,\eta_{n}^{\psi }=0.0001,\eta_{n}^{r}=0.0001$ and the batch size is 64. The step size for Projection Algorithm is $\epsilon_{n}=n^{-0.95}$. Figure 5 plots the errors for the state-dependent $\rho_{i}=2-\frac{3}{2N-4}\left(i-1\right)$. We set the initial value $\theta_{0}^{i}=8+\frac{35}{250}\left(i-1\right)$ for i∈{1,2,…,250}, $\theta_{0}^{251}=44$, $\theta_{0}^{i}=43$ for i∈{252,…,305}, $\theta_{0}^{306}=48$, $\theta_{0}^{307}=42$ and $\theta_{0}^{i}=43-\frac{38}{293}\left(i-1\right)$ for i∈{308,309,…,499}, $\psi_{0}=\left[0,0,\ldots ,0\right],r_{0}=0$ and the learning rate is $\eta_{n}^{\theta }=0.0002$, $\eta_{n}^{\psi }=0.0001,\eta_{n}^{R}=0.0001$ with the batch size as 128. The step size for the Projection Algorithm is $\epsilon_{n}=n^{-0.95}$. Both figures demonstrate that actor-critic algorithm performs quite well on this example.

In Table 1, we compared the CPU time of each algorithm in the M/M/1/500 queue when they obtain an accuracy at $2\times 10^{-1}$. We found that our algorithm cost less time on this example.

## 5. Summary and Conclusions

In this paper, we propose a reinforcement learning (RL) method for quasi-stationary distribution (QSD) in discrete time finite-state Markov chains. By minimizing the KL-divergence of two Markovian path distributions induced by the candidate distribution and the true target distribution, we introduce the formulation in terms of RL and derive the corresponding policy gradient theorem. We devise an actor-critic algorithm to learn the QSD in its parameterized form $\alpha_{\theta }$. This formulation of RL can receive benefit from the development of the RL method and the optimization theory. We illustrated our actor-critic methods on two numerical examples by using simple tabular parametrization and gradient descent optimization. It has been observed that the performance of our method is more prominent for large scale problems.

We only demonstrate the preliminary mechanism of the idea here, and there is much space left for improving the efficiency and extensions in future works. The generalization from the current consideration of finite-state Markov chain to the jump Markov process and the diffusion case is in consideration. More importantly, for very large or high dimensional state space, modern function approximation methods such as kernel methods or neural networks should be used for the distribution $\alpha_{\theta }$ and the value function $V_{\psi }$. The recent tremendous advancement of optimization techniques for policy gradient in reinforcement learning could also contribute much to efficiency improvement of our current formulation.

## Figures and Tables

**Table 1:** The CPU time of each algorithm in the M/M/1/500 queue when they obtain the accuracy at $2\times 10^{-1}$.

Algorithm	Vanilla	Projection	Polyak Averaging	ac_α
Time (s)	1038.3279	429.6304	505.2299	186.9280
Time (s)	753.9503	259.0671	268.5476	251.5370

## Data Availability

No new data were created or analyzed in this study. Data sharing is not applicable to this article.

## Appendix A

In this appendix, we discuss the computation of the gradient of $\nabla_{\theta }\ln K_{\alpha_{\theta }}$ and $\nabla_{\theta }\ln K_{\beta_{\theta }}$. Note that $\nabla_{\theta }\alpha_{\theta }$ is straightforward since we model α in its parametrization form θ. By definition (4), we have the following:

$$\nabla_{\theta }\ln K_{\alpha_{\theta }}\left(X_{t},X_{t+1}\right)=\frac{1-K(X_{t},E)}{K_{\alpha_{\theta }}\left(X_{t},X_{t+1}\right)}\nabla_{\theta }\alpha_{\theta }\left(X_{t+1}\right)$$

and the following as well:

$$\nabla_{\theta }\ln K_{\beta_{\theta }}\left(X_{t},X_{t+1}\right)=\frac{1-K(X_{t},E)}{K_{\beta_{\theta }}\left(X_{t},X_{t+1}\right)}\nabla \beta_{\theta }\left(X_{t+1}\right).$$

where $K_{\beta }\left(x,y\right)=K\left(x,y\right)+\left(1-K\left(x,E\right)\right)\beta \left(y\right)$. The vector K(x,E) for any *x* can be pre-computed and saved in tabular form.

By (19), the one-step distribution β is computed below.

$$\beta \left(X_{t+1}\right)=\sum_{x}\alpha \left(x\right)\left[K\left(x,X_{t+1}\right)+\left(1-K\left(x,E\right)\right)\alpha \left(X_{t+1}\right)\right]\approx \frac{1}{n}\sum_{i=1}^{n}K\left(Z_{i},X_{t+1}\right)+\left(1-K\left(Z_{i},E\right)\right)\alpha \left(X_{t+1}\right)$$

Here, the samples $Z_{i}∼\alpha$ could be approximated by stationary distribution μ; thus, one may simply use the known sample $X_{t}$ to replace $Z_{i}$ with n=1.

To find $\nabla_{\theta }\beta_{\theta }$, we use stochastic approximation again.

$$\begin{array}{cc} \nabla_{\theta }\beta_{\theta }\left(X_{t+1}\right) & =\sum_{x}\nabla \alpha_{\theta }\left(x\right)\left[K\left(x,X_{t+1}\right)+\left(1-K\left(x,E\right)\right)\alpha_{\theta }\left(X_{t+1}\right)\right]+\left[\sum_{x}\alpha_{\theta }\left(x\right)\left(1-K\left(x,E\right)\right)\right]\nabla_{\theta }\alpha_{\theta }\left(y\right),\\ & \approx \frac{1}{n}\sum_{i=1}^{n}\nabla \ln\alpha_{\theta }\left(Z_{i}\right)\left[K\left(Z_{i},X_{t+1}\right)+\left(1-K\left(Z_{i},E\right)\right)\alpha_{\theta }\left(X_{t+1}\right)\right]+\left(1-K\left(Z_{i},E\right)\right)\nabla_{\theta }\alpha_{\theta }\left(X_{t+1}\right). \end{array}$$
